# Efficacy of cognitive behavioral therapy for anxiety and depression in Parkinson’s disease patients: an updated systematic review and meta-analysis

**DOI:** 10.1007/s10072-024-07659-6

**Published:** 2024-07-03

**Authors:** Asmaa Zakria Alnajjar, Moaz Elsayed Abouelmagd, Abdulrahman Krayim, Maickel AbdelMeseh, Nagham Bushara, Yehia Nabil

**Affiliations:** 1 Faculty of Medicine, Al-Azhar University, Gaza, Palestine; 2 Faculty of Medicine, Cairo University, Cairo, Egypt; 3 Faculty of Medicine, Al-Azhar University, Cairo, Egypt; 4 Faculty of Medicine, Alexandria University, Alexandria, Egypt; 5 Faculty of Medicine, Zagazig University, Zagazig, Egypt

**Keywords:** Anxiety, Depression, Cognitive-behavioral therapy, Parkinson’s Disease

## Abstract

**Background:**

Parkinson’s disease (PD) patients often experience non-motor symptoms like depression and anxiety, significantly impacting their quality of life. With the limited effectiveness of pharmacological treatments, effective non-pharmacological interventions are needed. This systematic review and meta-analysis aimed to evaluate the efficacy of cognitive-behavioral therapy (CBT) in reducing depression and anxiety symptoms in PD patients.

**Methods:**

Randomized controlled trials (RCTs) exploring CBT's effectiveness for depression and anxiety in PD patients were included. Studies published until April 2023 were identified from PubMed, Web of Science, and Scopus. Methodological quality was assessed using the Risk of Bias-2 (ROB-2) tool. Statistical analysis involved calculating the standardized mean difference (SMD) and corresponding 95% confidence intervals (CIs) using Review Manager 5.4.1.

**Results:**

The systematic review included 12 studies involving 241 PD patients. CBT led to a substantial reduction in anxiety (SMD -0.95, 95% CI [-1.15 to -0.74], *P* < 0.00001) and depression (SMD -1.02, 95% CI [-1.39 to -0.65], *P* < 0.0001). Both traditional CBT and tele-CBT (administered over the phone or internet) were effective in treating depression and anxiety. Traditional CBT improved depression (SMD -1.16, 95% CI [-1.83 to -0.49], *P* < 0.00001), while tele-CBT showed comparable results (SMD -0.90, 95% CI [-1.31 to -0.48], *P* < 0.00001). For anxiety, both traditional CBT (SMD -0.94, 95% CI [-1.25 to -0.63], *P* < 0.00001) and tele-CBT (SMD -0.95, 95% CI [-1.22 to -0.67], *P* < 0.00001) significantly reduced symptoms.

In conclusion, this systematic review and meta-analysis demonstrated the efficacy of CBT in reducing depression and anxiety in PD patients. Healthcare providers are encouraged to integrate CBT into their treatment protocols. However, additional high-quality studies with longer-term follow-up assessments are needed to further enhance understanding in this area.

**Prospero Registration:**

CRD42023424758.

**Supplementary Information:**

The online version contains supplementary material available at 10.1007/s10072-024-07659-6.

## Background

Parkinson's disease (PD) is the second most prevalent neurodegenerative disorder and affects a significant number of individuals worldwide [1, 2]. While PD is commonly associated with motor symptoms, there are also prevalent nonmotor symptoms, including emotional and behavioral disturbances, that profoundly impact daily functioning and quality of life. Among these, depression and anxiety are the most common psychological disturbances and have been identified as stronger predictors of poor quality of life in PD patients [3, 4].

Depressive symptoms exist in 40–50% of PD patients and can manifest at any stage of disease progression [5]. Unfortunately, anxiety and depression are often underdiagnosed and undertreated in this population [4]. Although there are evidence-based therapies for these conditions, few specifically target individuals with PD. Furthermore, the limited evidence for effective pharmacological treatment of anxiety in PD patients, coupled with concerns about potential side effects and complications, restricts the use of psychiatric medications among elderly individuals, including PD patients [6].

Cognitive behavioral therapy (CBT), a form of talk therapy that focuses on identifying and modifying negative thoughts, patterns, and behaviors, has shown promise in treating mental health disorders such as anxiety and depression. Preliminary research, including case studies and uncontrolled trials, has provided early efficacy support for CBT in PD patients [7]. Additionally, the use of multimodal approaches, including telephone and video conferencing, has been explored as alternatives to face-to-face therapy sessions, overcoming barriers to engagement in telehealth therapy [8, 9]. Moreover, recent studies have suggested the potential cost-effectiveness of CBT in PD patients [10].

Previous systematic reviews have addressed the efficacy of CBT on anxiety and depression in PD patients [11, 12]. However, since they were published more studies with large samples have been published which may change the results of the previous meta-analysis. This study aims to fill the gap in the current understanding of treatment strategies for PD patients by assessing the impact of CBT on depression and anxiety in PD patients. In addition, we aim to explore the differences between traditional face-to-face CBT compared to tele-CBT approaches.

## Methods

In this study, we adhered to the guidelines outlined in the Preferred Reporting Items for Systematic Reviews and Meta-Analyses (PRISMA) and in strict accordance with the Cochrane Handbook of Systematic Reviews and Meta-analysis. Additionally, the protocol was registered in the International Prospective Register of Systematic Reviews (PROSPERO), with the register number: CRD42023424758.

### Eligibility criteria

Studies that met the following criteria were included: 1) were randomized controlled trials (RCTs); 2) included participants aged 18 years or older who were clinically diagnosed with PD; 3) had Parkinson’s disease (PD) with depression or anxiety diagnosed by a psychiatrist or a validated tool; 4) had CBT and derivative therapy; and 5) had outcomes assessed using validated measurement tools; 6) studies with a comparison group receiving non-CBT interventions such as usual care, waiting list controls, or clinical monitoring only.

We excluded studies that met the following conditions: (1) had a significant physical impairment, acute suicidal ideation, psychosis, drug misuse, or serious dementia; (2) lacked data or insufficient data; (3) had duplicate publications of the same patient dataset; and (4) lacked accessible full text in the English language.

### Information source and search strategy

A comprehensive and systematic search was conducted across major electronic databases, namely, MEDLINE via PubMed, Web of Science, and Scopus.

The employed search strategy consisted of a combination of keywords and controlled vocabulary terms to retrieve relevant articles. The following search strategy was used for PubMed: [("Cognitive‒Behavioral Therapy" OR "cognitive behavioral therapy" OR "CBT" OR "psychotherapy" OR "cognitive therapy") AND ("Parkinson's disease" OR "Parkinson disease" OR "functional disability") AND ("anxiety" OR "depression")]. The search was conducted from the inception of each database until April 2023.

All the search records obtained from each database were consolidated into EndNote reference management software version 21 to identify and remove duplicates.

### Selection of studies and data collection

The selection of appropriate papers for our research project involved all the authors and consisted of two distinct screening phases. During the first phase, the titles and abstracts of each article were evaluated independently by two groups of authors. This initial assessment was repeated for accuracy. Subsequently, for articles that successfully passed the first phase, a full-text screening was conducted to identify relevant studies. The data extraction was conducted by all reviewers using a standardized online data collection form. Any disagreements were resolved through discussion and by consulting Alnajjar A.Z.

### Risk of *bias* assessment

All authors employed the Cochrane Risk of Bias 2 (ROB2) tool [13] to thoroughly assess potential bias in the selected studies. The ROB2 tool comprises five critical factors randomization process, deviations from intended interventions, missing outcome data, measurement of the outcome, and selection of the reported result that influenced the evaluation of bias.

### Measures of the effect of CBT and statistical analysis

The effect sizes of the studies in our meta-analysis were expressed as continuous outcomes using the mean change from baseline and its standard deviation (SD). The mean change in each included effect size was calculated by subtracting the baseline mean from the final mean, while the SD of change was imputed from the baseline and final SD and by a correlation coefficient (r) of 0.7 [14] calculated from studies with complete data.

The pooled effect estimate for primary outcomes was expressed as the standardized mean difference (SMD) of change with a 95% confidence interval. Effect sizes were pooled by either a fixed or random effect model according to heterogeneity using Review Manager version 5.4.1.

$$\begin{array}{l}Mean\;Change = Mean\;Final - Mean\;Baseline\\ SDchange= \sqrt{S{D}^{2}baseline + S{D}^{2}final - \left(2 \times r \times SDbaseline \times SDfinal\right)}\end{array}$$

### Assessment of heterogeneity

Heterogeneity between studies was assessed through visual inspection of forest plots, the chi-square test, and the I2 test for the level of heterogeneity due to heterogeneity other than chance. I2 values lower than 50% were considered insignificant heterogeneity, and I2 values ≥ 50% were considered substantial heterogeneity. When heterogeneity was absent or nonsignificant, a fixed effect model and inverse variance method with 95% confidence intervals (CIs) were used to pool estimates. However, when significant heterogeneity was observed, the random effect model Der Simonian-Laird (with 95% CI) was used to account for within- and between-study heterogeneity.

### Publication *bias*

We visually analyzed the funnel plots of the meta-analysis models for the potential influence of publication bias. An unequal distribution of studies around the pooled effect estimate could suggest the presence of publication bias. Sterne et al. proposed that funnel plot asymmetry be based on a minimum of 10 studies to serve as an effective tool for detecting publication bias within the context of meta-analysis [15]. Additionally, we used Egger’s test, and a P value < 0.5 was considered significant publication bias.

## Results

### Study selection

**Fig. 1:**
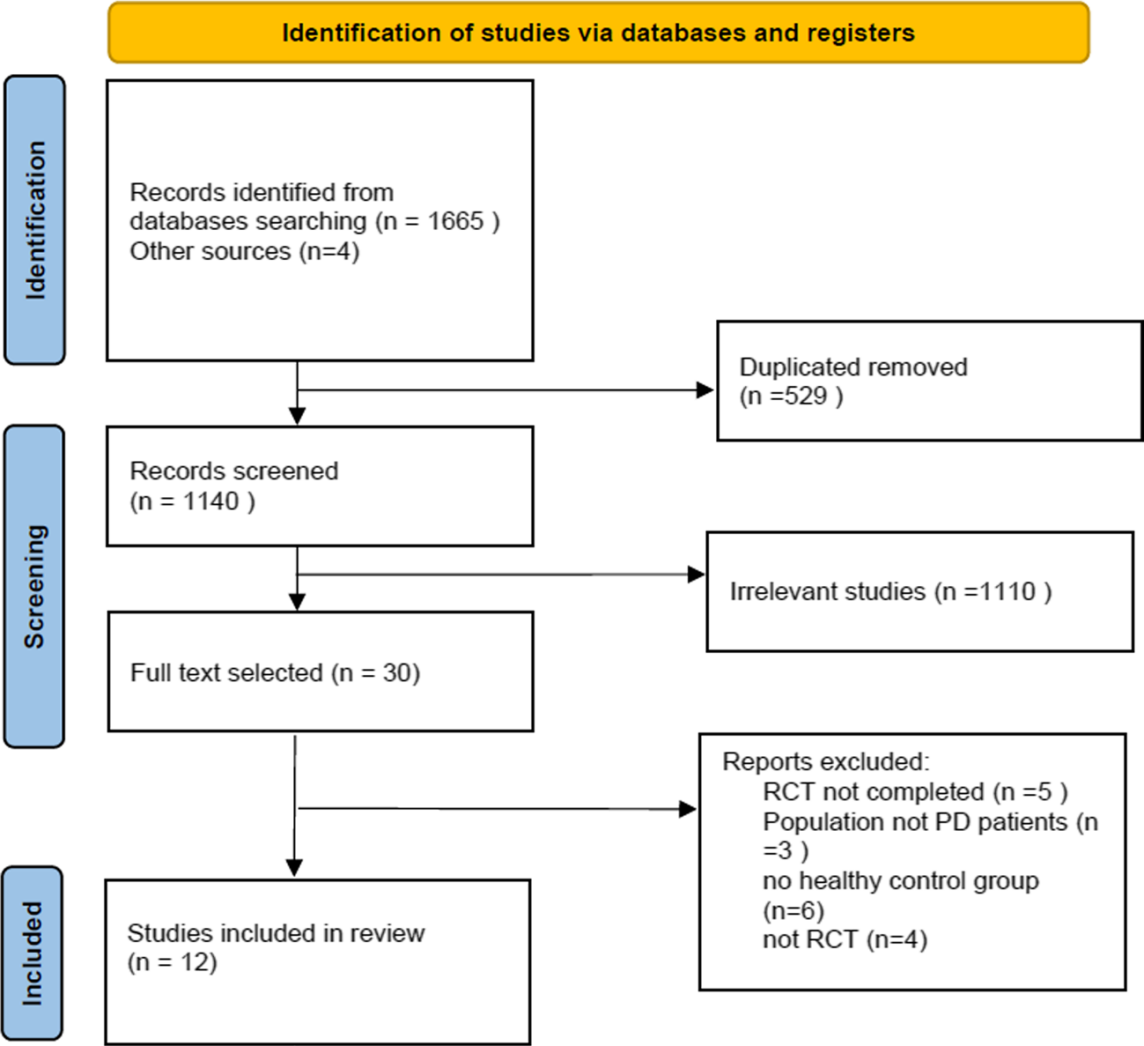
PRISMA Flow chart of the study selection process

Initial searches through databases and references yielded 1671, of which 529 were removed as duplicates. After screening the titles and abstracts of 1140 studies, only 30 met our eligibility criteria. Additionally, 18 studies were excluded after a full-text screening for reasons stated in the PRISMA diagram. Finally, twelve studies [16, 17] were included in our systematic review and meta-analysis. Figure 1 presents the PRISMA flow diagram of the identification and selection of studies.

### Study characteristics

The sample sizes ranged from 11 to 90 participants, resulting in a total of 467 PD patients. The studies were conducted in different regions, with seven studies conducted in North America [16, 18–23], two in Australia [23, 24], two in Europe [25, 26], and one in Asia [27]. Table 1 presents an overview of the key characteristics of the eleven studies [16, 18–27] included in the analysis.

**Table 1:** Characteristics of the Included Studies

Study ID	Study design	Country	Inclusion and exclusion criteria	Number of participants	Age (years) Mean ± Standard deviation	Disease Duration	Intervention (Type, duration)	Number and duration of sessions	Follow up duration (weeks)	Outcome/measurement of the outcome and their effect on Anxiety and depression
Bae et al. 2015 [27]	Randomized controlled trial	Korea	IPD is diagnosed by neurologist recruited from a clinic without dementia	T:9 C:23	T: 60.9 ± 6.13 C: 68.3 ± 7.15	N/A	T: CBT, 8 wk C: Usual Care	8 weeks, 60–70 min/session	4	Anxiety: STAI Depression: BDI
Dobkin et al 2011 [18]	Randomized controlled trial	USA	PD patients recruited from a clinic with CGI ≥ 4 w, age 35–85 without dementia	T: 41 C: 39	T: 63.73 C: 65.44	T: 6.53 C: 6.13	T: CBT, 10 wk C: clinical monitoring only	10 sessions over 10 weeks; 60–75 min duration	4	Anxiety: HAM-A, Depression: HAM-D, BDI
Moonen et al. 2021 [24]	Randomized Controlled Trial	The Netherlands	IPD patients diagnosed with QSBB and recruited from a clinic irrespective of their disease stage without severe psychiatric conditions	T: 24 C: 24	T: 63.3 ± 7.2 C: 63.3 ± 8.4	T: 7.4 ± 5.6 C: 4.7 ± 4.0	T: CBT, 10 wk C: clinical monitoring only	10 weekly sessions, the duration of an individual session is 60–75 min	12	Anxiety: HARS, PAS
Rios et al. 2013 [22]	Randomized pilot study	Canda	IPD patients with insomnia more than 6 months in a university hospital	T:6 C:6	T: 64.5 ± 16.3 C: 69.5 ± 10.3	T: 5.2 ± 1.8 C: 5.2 ± 4.4	T: CBT, 6 wk., bright light, and sleep hygiene training therapy C: Bright light therapy	6 weekly sessions of 90 min	N/A	Depression: BDI
Troeung et al 2014 [26]	waitlist-controlled trial design	Australia	PD patients of at least 6 months period from registers and hospitalswithout dementia, psychosis, or uncontrolled bipolar disorder	T: 11 C: 7	T: 68 ± 7.72 C: 62 ± 8.34	T: 5.70 ± 5.50 C: 4.29 ± 3.15	T: CBT, 8 wk C: waiting list	120 min each, once/week,8 weeks	(4, 24) *	Anxiety: DASS Depression: DASS
Wuthrich et al. 2018 [25]	Randomized controlled trial	Australia	PD patients over 50 recruited through flyers in a PD newspaper without dementia, psychosis, or uncontrolled bipolar disorder	T:6 C: 5	68.82 ± 9.35	N/A	T: CBT, 10 wk C: waitlist	10 weekly sessions, each one 45 min	N/A	Anxiety: DASS Depression: DASS
Kraepelien et al. 2020 [21]	Randomized Controlled Trial	Sweden	PD patients with significant self-reported problems (WSAS > 17) without dementia, psychosis, or uncontrolled bipolar disorder	T:38 C:39	T: 65.9 ± 8.5 C: 66.1 ± 9.8	T: 8.3 ± 4.4 C: 9.6 ± 5.7	T: CBT, 10 wk C: standard medical treatment plus being on waitlist	10 weeks	N/A	Anxiety: HADS-A Depression: HADS-D
Okai et al 2013 [17]	Randomized controlled trial	Britain	IPD according to QSBB from a hospital without dementia or significant cognitive impairment	T: 28 C: 17	T: 59.3 ± 6 8.1 C: 57.9 ± 6 9.5	T: 10.5 ± 66.0 C: 8.8 ± 65.6	T: CBT, 12 wks C: Waiting list	12 sessions,12 weeks	12	Anxiety: BAI, Depression: BDI,
Piers et al 2022 [19]	Pilot Randomized Controlled Trial	USA	IPD patients identified through medical records without dementia or significant cognitive impairment	T:6 C:6	T: 58.5 ± 8.1 C: 64.1 ± 5.9	T: 7.9 ± 2.8 C: 8.8 ± 5.1	T: CBT, 12 wks C: waitlist	12 weekly 50- to 60-min sessions	6*	Anxiety: BAI Depression: BDI-II
Calleo et al 2015 [16]	Pilot Study	USA	IPD patients from PD centers without dementia, psychosis, or uncontrolled bipolar disorder	T: 7 C: 4	62.9 ± 7.3	N/A	T: CBT, 8 wks C: Usual Care	8 sessions over 12 weeks; 30–40 min duration	4	Anxiety: HAM-A Depression: HAM-D
Patel et al 2017 [20]	pilot trial	USA	PD patients from a clinic with ISI > 11 diagnosed by neurologist without dementia or psychosis	T:14 C:14	T: 63.1 ± 6.8 C: 64.7 ± 9.5	N/A	T: CBT, 6 wk C: Usual Care	6 sessions over 6 weeks	N/A	Anxiety: GAI Depression: PHD-9
Dobkin 2021 [23]	Randomized Controlled Trial	USA	PD patients with medical records without marked cognitive impairment, bipolar or psychotic disorders	T:45 C:45	T:67.27 ± 7.79 C:66.42 ± 9.51	T: 5.4 ± 5.011 C: 5.24 ± 5.13	T: CBT, 10 wk C: TAU	60 min each, once/week	24	Anxiety: HAMA Depression: HAMD

*T* intervention group, *C* control group, *PD* Parkinson’s disease, *CBT* Cognitive Behavioral Therapy, *wk* weeks, *USA* United States of America, *NA* Not available, *CGI *Clinical Global Impression Severity Scale, *HAM-D* Hamilton Rating Scale for Depression, *HAM-A* Hamilton Rating Scale for Anxiety, *DASS-D* Anxiety and Stress Scale-Depression, *DASS-A* Anxiety and Stress Scale-Anxiety, *BAI* Beck Anxiety Inventory, *BDI* Beck Depression Inventory, *GAI* Geriatric Anxiety Inventory, *PHD-9 *Patient Health Questionnaire, *STAI* the State-Trait Anxiety Inventory, *HARS* the Hamilton Anxiety Rating Scale, and *PAS* the Parkinson Anxiety Scale, *TAU* treatment as usual, *DSM-IV* Diagnostic and Statistical Manual of Mental Disorders, fourth edition, *GDS* Geriatric Depression Scale, *IPD* idiopathic Parkinson's disease, *QSBB* Queens Square Brain Bank diagnostic criteria, *WSAS* Work and Social Adjustment Scale

*studies conducted follow-up evaluation for the intervention group only and were not included in the subgroup analysis

CBT was administered through diverse approaches, including internet-based and telephone-based methods. The studies employed various comparators, with four utilizing telephone/internet sessions [19–21, 23, 27] and seven employing face-to-face sessions [16–18, 22–27]. The primary outcome of interest was depression, which was assessed in ten studies using tools such as the Hamilton Rating Scale for Depression (HAM-D), the Depression, Anxiety, and Stress Scale-Depression (DASS-D), and the Patient Health Questionnaire (PHQ-9). Anxiety was assessed in nine studies employing measures such as the Hamilton Rating Scale for Anxiety (HAM-A), the Depression, Anxiety, and Stress Scale-Anxiety (DASS-A), and the Geriatric Anxiety Inventory (GAI).

### Risk of *bias* in studies

**Fig. 2:**
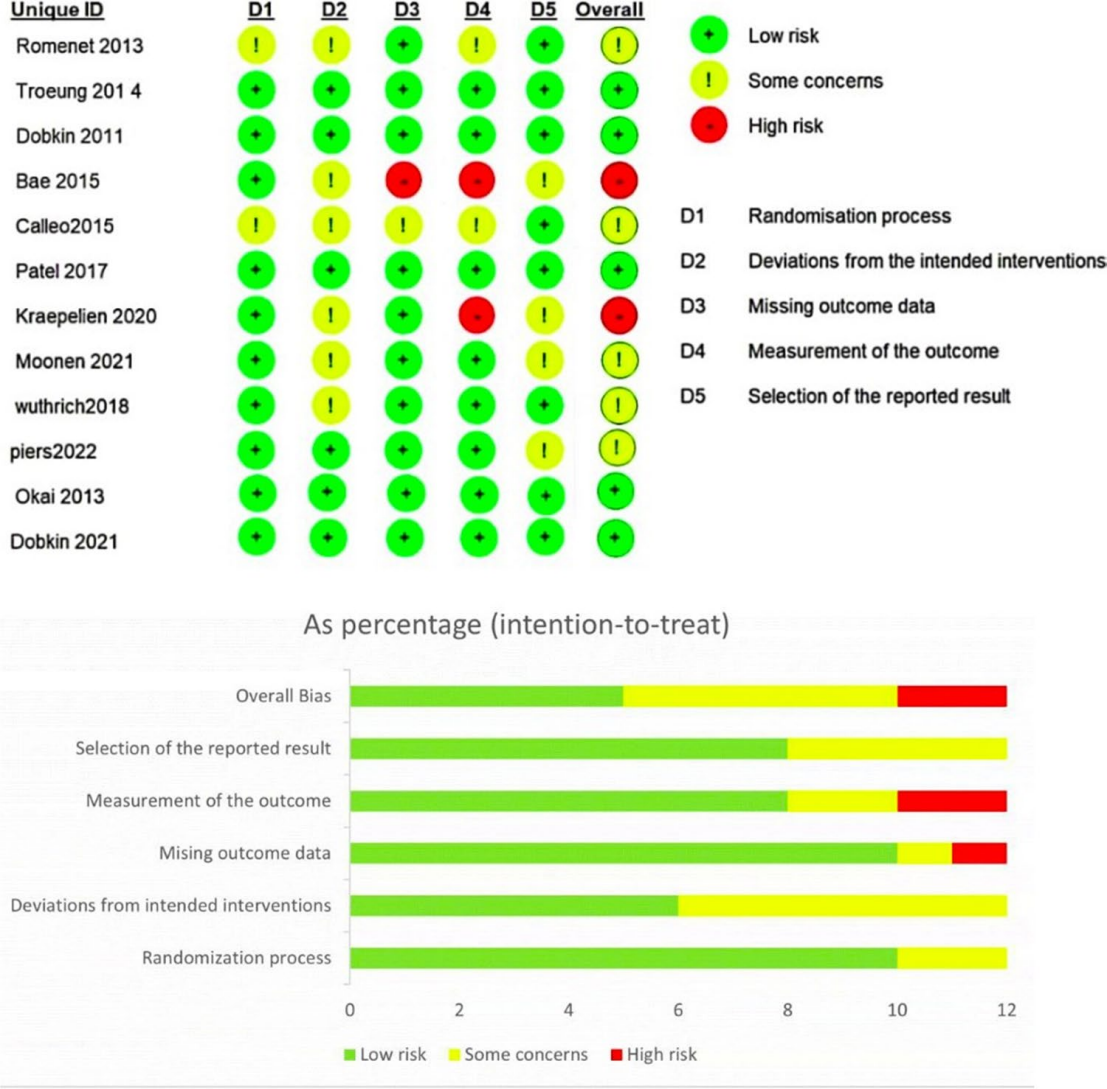
Assessment of the Methodological Quality of the Included Trials, Evaluated Using the Cochrane Risk-of-Bias Tool (v. 2.0). Risk of bias graph (downward)) represents the percentage of each bias level for five items. Risk of bias summary (upward), represents the level of specific items in each study. Different colors (green, red, yellow) and symbols (“ + ”, “—“, “?”) indicate “low risk of bias”, “high risk of bias” and “unclear risk of bias”. A study with three or more green “ + ” can be considered to have a low risk of bias

The methodological quality assessments of the eligible RCTs are shown in Fig. 2 and ranged from low to high risk. We found that ten studies provided sufficient detail on the randomization process, indicating a low risk of bias in this aspect. Additionally, six studies showed no deviation from the intended interventions, indicating a low risk of bias in this area. However, Bae 2015 et al. had missing outcome data, which could lead to a high risk of bias in this aspect. Moreover, Kraepelin 2020 et al. and Bae 2015 et al. were assessed to have a high risk of bias regarding the measurement of outcomes. On the other hand, eight studies had a low risk of bias concerning the selection of reported results.

The overall assessment of the 12 studies revealed that five of them were classified as having a low risk of bias, and five raised some concerns regarding their risk of bias.

### Impact of CBT on depression

**Fig. 3:**
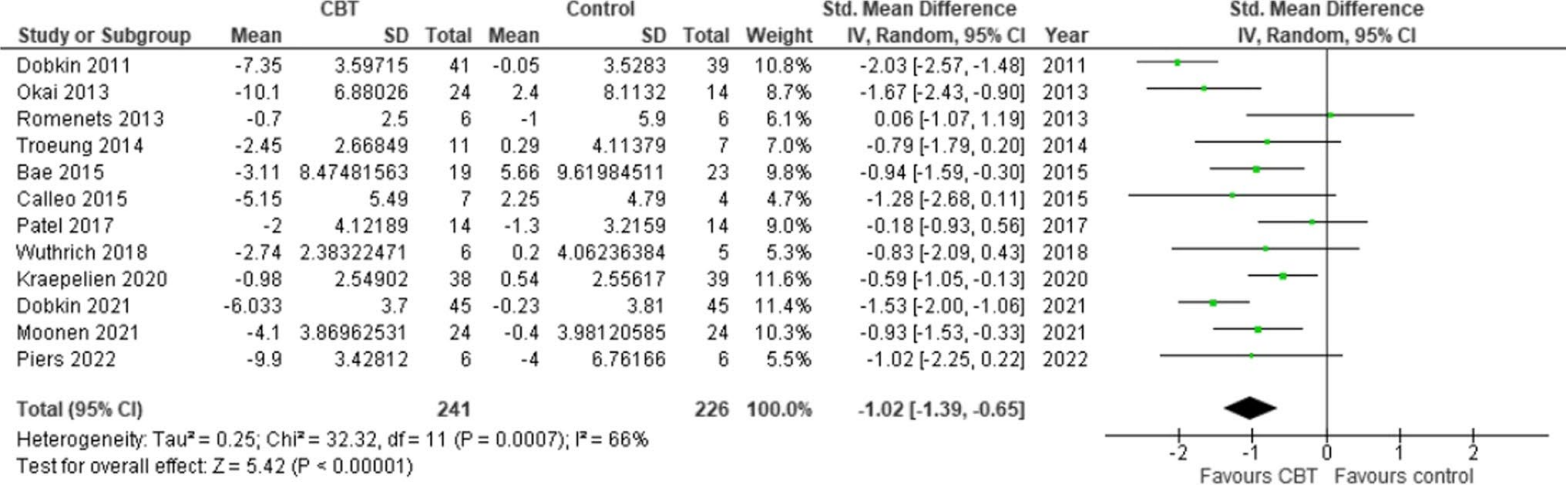
Forest plot of the Impact of CBT on Depression

Figure 3 shows that the effects of CBT on depression were assessed using BDI, the Hamilton Depression Rating Scale (HDRS), the Hospital Anxiety and Depression Scale (HADS), DASS-D, and the Geriatric Depression Scale (GDS). Twelve studies (N CBT = 241, N control = 226) were included in the pooled effect estimate analysis to assess the effect of CBT on depression in PD patients (Fig. 3). The results showed a significant and large positive effect of CBT intervention on depression (SMD = -1.02, 95% CI: [-1.39 to -0.65]; *p* < 0.00001). Significant heterogeneity was present (*P* = 0.0007; I2 = 66%), so the random effects model was used.

#### Effect of traditional CBT vs Tele-CBT on depression

A subgroup analysis was performed based on the type of CBT strategies employed in the studies (Supplementary material. 1). The two categories of CBT strategies were traditional CBT, which consisted of face-to-face sessions with psychiatrists, and tele-CBT, which involved telephone and internet sessions, tasks, instructions, and online reading through a dedicated platform. The test of subgroup differences indicated that there was no statistically significant difference between the subgroups (*P* = 0.51). However, fewer studies were included in the traditional CBT group than in the tele-CBT group. The pooled effect sizes for both groups were significant, with Tele-CBT (SMD -0.90, 95% CI [-1.31 to -0.48], *P* < 0.00001) and Traditional CBT (SMD -1.16, 95% CI: [-1.83, − -0.49], *P* < 0.00001), indicating that both types of therapy are efficient treatments for depression.

#### Sensitivity analysis

The sensitivity analysis was conducted by the exclusion method, and the studies by Dobkin et al. (2011) and 2021 were the primary sources of heterogeneity. A meta-analysis was also conducted after the exclusion of studies resulting in a decrease in heterogeneity to a nonsignificant level (*P* = 0.24, I2 = 22%). Although the effect size decreased, it remained large and significant (SMD = -0.79, 95% CI: [-1.03, -0.54]; *P* < 0.00001), providing evidence that the results of our study are robust. The studies by Dobkin et al. were the largest, included 150 PD patients, and had a low risk of bias. The greater effect sizes observed may be related to the CBT program, which was tailored to PD patients' needs and their caregivers (Supplementary material. 2).

#### Publication bias for depression

**Fig. 4:**
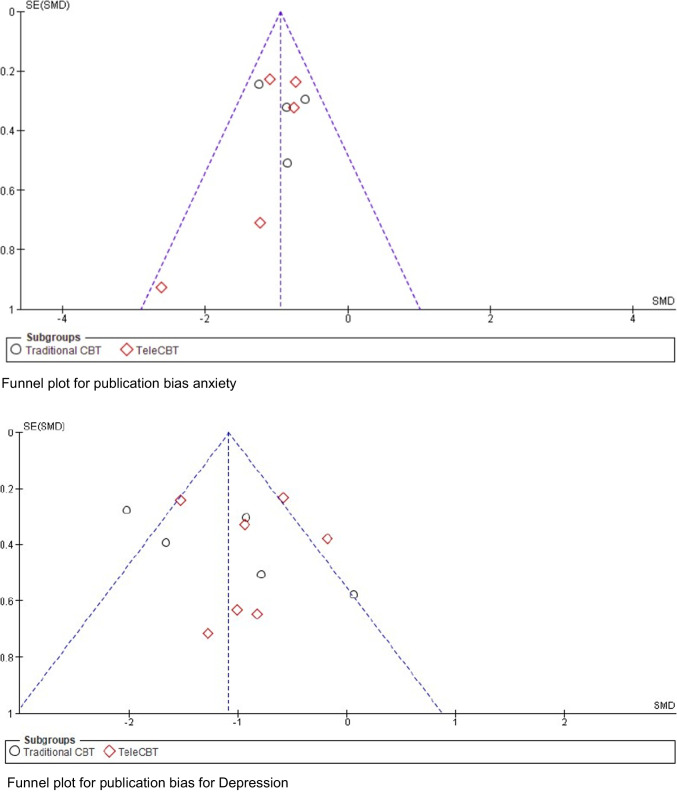
Funnel plots for publication bias of the effect of CBT on depression (on the left) and anxiety (on the right)

A funnel plot was generated to visually evaluate the presence of publication bias, and no bias was found (Fig. 4). Egger's test indicated no significant publication bias (*P* = 0.4590) for depression.

### Impact of CBT on anxiety

**Fig. 5:**
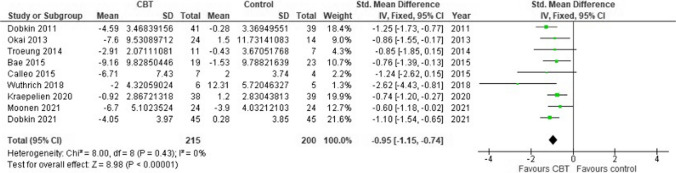
Forest plot of the Impact of CBT on Anxiety

Anxiety was assessed in the included studies BAI, HAMA, HADS, DASS-A, and GAI. Nine studies were included in this meta-analysis, which included a total of 415 PD patients. The results demonstrated a large and significant positive effect of CBT in treating anxiety (SMD = -0.95, 95% CI: [-1.15, − -0.74]; *P* < 0.00001), with no significant heterogeneity detected between studies (*P* = 0.43; I2 = 0%), and a fixed effects model was used on that basis Fig. 5

#### Effect of traditional CBT vs. Tele CBT on anxiety

A subgroup analysis was performed based on the type of CBT strategies employed in the studies (supplementary material. 3). The test of subgroup differences indicated that there was no statistically significant subgroup effect (*P* = 0.99), indicating that the type of CBT does not modify the effect of CBT compared to the control on anxiety in PD patients. However, fewer studies were included in the traditional CBT group than in the tele-CBT group. The pooled effect sizes for both groups were very close where the pooled effect size of traditional CBT was (SMD = -0.94, 95% CI: [-1.25, − -0.63]; *P* < 0.00001) and tele-CBT was (SMD = -0.95, 95% CI: [-1.22, − -0.67]; *P* < 0.00001), indicating that both types of therapy are efficient treatments for anxiety.

#### Publication bias for anxiety

A funnel plot was generated to visually evaluate the presence of publication bias, and no bias was found (Fig. 4). Egger's test indicated no significant publication bias (*P* = 0.1242) for Anxiety.

### Follow-up analysis

Six studies conducted a follow-up evaluation of participants' depression and anxiety scores after the end of treatment. Among these studies, three had a follow-up period of one month, one had three months, and two had two months. The pooled effect estimates of CBT on depression and anxiety after follow-up were of medium size and statistically significant (SMD = -0.67, 95% CI: [-1.01, -0.31], *P* = 0.0001) and (SMD = -0.59, 95% CI: [-0.82, -0.37], *P* < 0.00001), respectively, indicating positive long-term effects of CBT on both depression and anxiety. The results of the subgroup analysis demonstrated larger effect sizes in the 6-month group than in the other groups, indicating that CBT may have persistent benefits (Supplementary material 0.4 and 0.5).

## Discussion

Our analysis demonstrated that CBT may be an effective treatment for depression and anxiety in patients with Parkinson’s disease. This finding is consistent with prior systematic reviews by Luo et al. [28], Hong et al. [11], and Zhang et al. [12]. Our study expands upon prior research by including additional RCTs and a more in-depth analysis of the effects of CBT on depression and anxiety.

The studies included in our analysis employed a diverse range of CBT modalities, session structures, and timeframes, which may have contributed to the variability among effect sizes reported in this study. Most studies favored individualized approaches rather than group sessions. Tele-CBT was conducted through various formats, such as telephone calls, video conferencing, and other electronic communication methods; however, other studies investigated face-to-face [17, 18, 22, 24, 26] therapy or a combination of both [25]. Additionally, email reminders and online applications were utilized to monitor patient progress, and some sessions were conducted either in patients' homes or in clinics, with some studies combining both settings. Most programs were administered by psychologists or individuals trained in CBT programs. Some of these CBT programs were specifically tailored to PD patients’ needs, providing them with problem-solving skills to modify negative thoughts and engage in behaviors necessary to cope with PD [29]. However, it should be noted that not all studies provided specific details regarding the programs and their administrators. This should be taken into consideration in future trials.

The duration of CBT programs included in our study varied from 6 to 12 weeks, with a frequency as high as once a day or once a week. Several studies have conducted follow-up assessments after the treatment period, with varying durations of follow-up ranging from one month [16, 18, 26, 27] to six months. For example, Moonen et al. (2018) conducted a three-month follow-up, while Dobkin et al. reported a six-month follow-up with a high patient retention rate of 78% [23]. The results of these studies suggest that CBT may have sustained benefits in reducing symptoms of depression and anxiety in PD patients over a longer period. However, more RCTs are needed to further explore the long-term effects of CBT among PD patients.

Regarding Depression, we analyzed twelve trials that involved a total of 467 participants. The studies included in our review used a variety of scales to measure depression, including the BDI, HAMD, HADS, and DASS. While the Moonen et al. study did not observe a significant difference between the two groups on the HAMD scale [24], other studies have reported the efficacy of CBT in reducing depression symptoms when using HAMD. Some studies argue that the BDI is a better diagnostic and rating tool than the HAMD because the BDI is less sensitive to motor symptoms and therefore can yield more accurate results [18, 30].

As for anxiety, several measurement tools, such as the BAI, HAMA, DASS, GAI, and GCI, were used to assess anxiety in the included studies. We relied primarily on the HAMA scale for evaluating anxiety levels. Some studies included doubts about HAMA as a scale for the measurement of anxiety in PD patients [21, 24]. Monen et al. reported a lack of efficacy of CBT on HAMA as their primary outcome while demonstrating a positive effect on the Parkinson's Anxiety Scale (PAS). The HAMA scale has been criticized for its insufficiency in distinguishing between anxiety and the effects of motor or depressive symptoms. However, HAMA is still the most widely used scale for assessing anxiety in PD patients. On the other hand, the PAS may represent a better assessment tool for anxiety in PD patients due to its insensitivity to motor and depressive symptoms [31]. The DASS-A and the GAI showed contradictory results in some studies where the GAI indicated a decrease in anxiety levels opposite to the DASS-A, indicating an exacerbation of anxiety symptoms. This highlights the need for standard tools for measuring anxiety and depression in further trials to confirm the robustness of our study.

Our study agrees with the results of previous systematic reviews [11, 12]. Hong et al*.*[11] included 8 RCTs with 247 patients and found large positive effects of CBT on anxiety and depression in PD patients. Zhang et al. [12] included 7 studies with 191 patients and found moderate effects of CBT on depression and anxiety in PD patients. Additionally, none of the previous systematic reviews investigated the effects of Tele-CBT vs traditional CBT or the effects of CBT on different follow-up points. we expand upon previous systematic reviews by including more RCTs and conducting subgroup analysis based on the type of CBT program (tele-CBT or traditional CBT) and follow-up periods. The High risk of bias in certain domains may affect the reliability of our findings. For instance, missing outcome data in Bae et al. (2015) could result in an incomplete understanding of the treatment's efficacy. Similarly, the high risk of bias in outcome measurement in Kraepelin et al. (2020) and Bae et al. (2015) could lead to inaccurate assessments of the treatment effects. 

The burden of caring for a PD patient can lead to psychological disturbances among caregivers, who also play a crucial role in the psychological health of PD patients. Several included studies [16, 18, 21, 24] have included caregivers in their treatment plans, either as a part of the treatment or as a target of CBT. According to Calleo et al*.,* four caregivers attended approximately 40% of the sessions supporting their family members, while according to Wuthrich et al*.,* three caregivers benefited from CBT sessions. On the other hand, Okai et al. reported no or a slight trend toward improvement in caregiver burden after involving caregivers in their CBT program. Therefore, further studies are necessary to investigate whether including caregivers in treatment plans benefits caregivers and patients.

The use of Tele-CBT has been increasing over the years to accommodate patients' needs. Tele-CBT interventions can overcome PD patients' barriers, such as motor disability, transportation limitations, and stigma [32]. Seven and five studies in our analysis demonstrated the effectiveness of Tele-CBT as a treatment for depression and anxiety, respectively. Furthermore. Our systematic review revealed no significant difference in efficacy between Tele-CBT and face-to-face CBT for PD [28]. In Calleo et al., 67% of patients preferred telephone sessions when provided with mixed models of Tele CBT and face-to-face CBT programs, which may alleviate concerns about the adaptations of elderly PD patients with technology. It is worth noting that Most of the included studies accounted for neurological or psychiatric comorbidities in their design. All of the studies in our review excluded dementia or severe cognitive impairment patients and many of them excluded patients with psychosis or uncontrolled bipolar disorder (see Table 1 for more details). 

CBT programs provide safe and efficient interventions for elderly patients who may not adhere properly to other treatments and medications [6]. Some studies included in our review reported completion rates of more than 80% [16, 21–26], reaching a completion rate of 95% for all sessions in Dobkin 2021. Most participants rated Tele CBT programs as helpful [16], easy to use [23] suitable for their mobility limitations [25], or excellent [21]. The most important skills learned were deep breathing, managing negative thoughts, and relaxation techniques [16, 23]. This finding indicates that CBT, especially tele-CBT programs, is an efficient solution for ensuring adherence in elderly people.

### Limitations

Despite adhering to meta-analysis guidelines, our study has limitations that must be acknowledged. First, the sample size was relatively small, potentially limiting the generalizability of our findings. Second, among the included studies, CBT delivery methods, duration of intervention, and outcome measures may have affected the consistency of the results. Third, the follow-up period was relatively short, suggesting the need for longer-term assessments to assess the long-term effects of CBT. Fourth, the lack of blinding in CBT interventions and the high risk of bias of some studies present a challenge to appropriate interpretation of the results. Finally, in our main analyses, we assessed the effects of interventions on the first endpoint, where some studies had their first endpoint after a period of intervention. This finding adds to the robustness of our study because the longer the follow-up period was, the lower the expected efficacy of the intervention. To demonstrate the efficacy of the intervention at different follow-up endpoints, we conducted subgroup analyses.

Recognizing and addressing these limitations in future research by incorporating larger sample sizes, utilizing randomized controlled trials, deciding on standard measurement tools and developing CBT programs tailored for PD patients, taking multiple measurements at different endpoints, and extending follow-up periods will enhance the robustness and applicability of our findings.

## Conclusion

Our findings support the efficacy of CBT in alleviating depression and anxiety symptoms in individuals with Parkinson's disease. tele-CBT approaches demonstrated comparable efficacy to face-to-face CBT, offering a promising solution to the barriers faced by PD patients in accessing psychological support. These findings emphasize the importance of integrating CBT into standard care for PD individuals experiencing depression and anxiety. However, to establish more robust evidence, future research should include larger-scale RCTs with longer follow-up periods to gain deeper insights into the long-term effects of CBT on depression and anxiety in this population.

## Supplementary Information

Below is the link to the electronic supplementary material.

Supplementary file1 (DOCX 1587 KB)

## Data Availability

The data supporting this study's findings are available from the corresponding author upon reasonable request. Data related to the current study are available from the corresponding author upon reasonable request.

## Declarations

### Guarantor

Moaz Elsayed Abouelmagd.

## Abbreviations

*CBT*: Cognitive behavioral therapy
*PD*: Parkinson’s disease
*RCT*: Randomized controlled trial
*PROSPERO*: Prospective Register of Systematic Reviews
*PRISMA*: Preferred Reporting Items for Systematic Reviews and Meta-Analyses
*HAM-D*: Hamilton Rating Scale for Depression
*HAM-A*: Hamilton Rating Scale for Anxiety
*DASS-D*: Anxiety and Stress Scale-Depression
*DASS-**A*: Anxiety and Stress Scale-Anxiety
*BAI*: Beck Anxiety Inventory
*BDI*: Beck Depression Inventory
*GAI*: Geriatric Anxiety Inventory
*PhD-9*: Patient Health Questionnaire
*STAI*: The State-Trait Anxiety Inventory
*HARS*: The Hamilton Anxiety Rating Scale
*PAS*: The Parkinson Anxiety Scale
