# Supplementary material for: Efficacy of cognitive behavioral therapy for anxiety and depression in Parkinson’s disease patients: an updated systematic review and meta-analysis
Source: Neurol Sci. 2024 Jul 3;45(11):5277–90. doi: 10.1007/s10072-024-07659-6 (PMC11470855; doi:10.1007/s10072-024-07659-6)
Supplement: Supplementary file 1 — Supplementary file1 (DOCX 1587 KB) [file 10072_2024_7659_MOESM1_ESM.docx]

# Supplementary material

**supplementary material .1:** Forest plot of the subgroup analysis of Effect of Traditional CBT vs Tele-CBT on Depression

**supplementary material .2**: Sensitivity analysis of the effect of CBT on depression

**supplementary material .3**: Forest plot of the subgroup analysis of the Effect of Traditional CBT vs Tele-CBT on Anxiety.

**supplementary material .4**: Sensitivity analysis of the effect of CBT on Anxiety

**supplementary material .5**:  Forest plot of the long-term effects of CBT on Depression versus control in PD patients based on follow-up values of different time points

**supplementary material .6**: Forest plot of the long-term effects of CBT on Anxiety versus control in PD patients based on follow-up values of different time points.


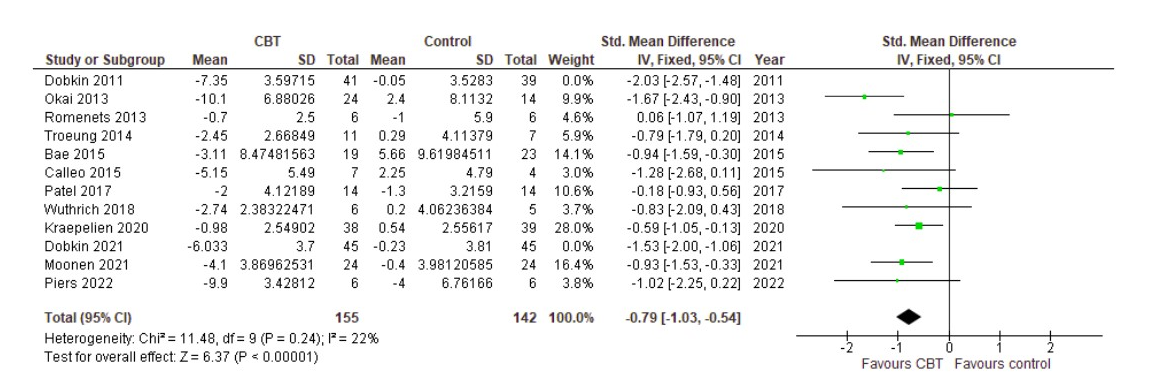

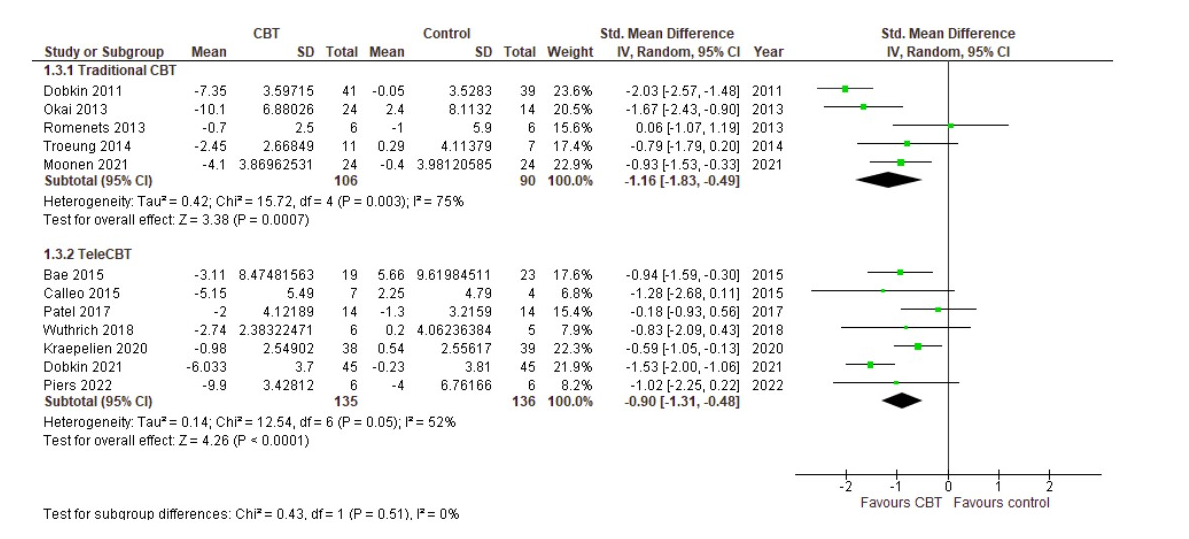
**supplementary material .1:** Forest plot of the subgroup analysis of Effect of Traditional CBT vs Tele-CBT on Depression.


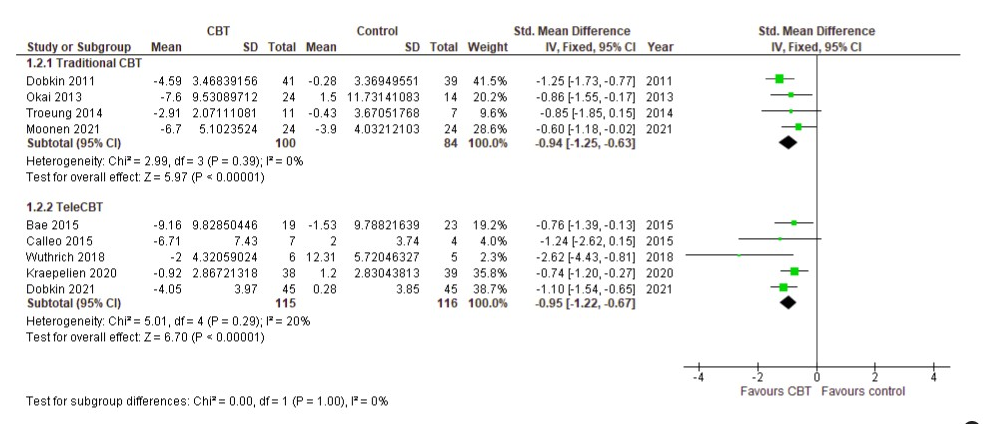
**supplementary material .2**: Sensitivity analysis of the effect of CBT on depression

**supplementary material .3**: Forest plot of the subgroup analysis of the Effect of Traditional CBT vs Tele-CBT on Anxiety.


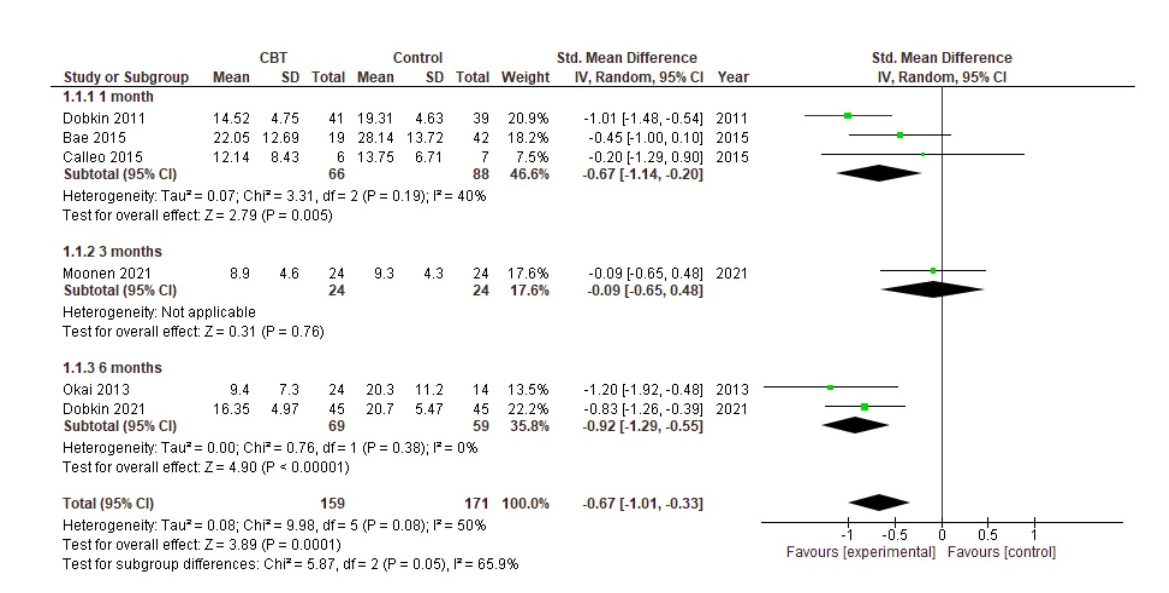


` **supplementary material .4**:  Forest plot of the long-term effects of CBT on Depression versus control in PD patients based on follow-up values of different time points


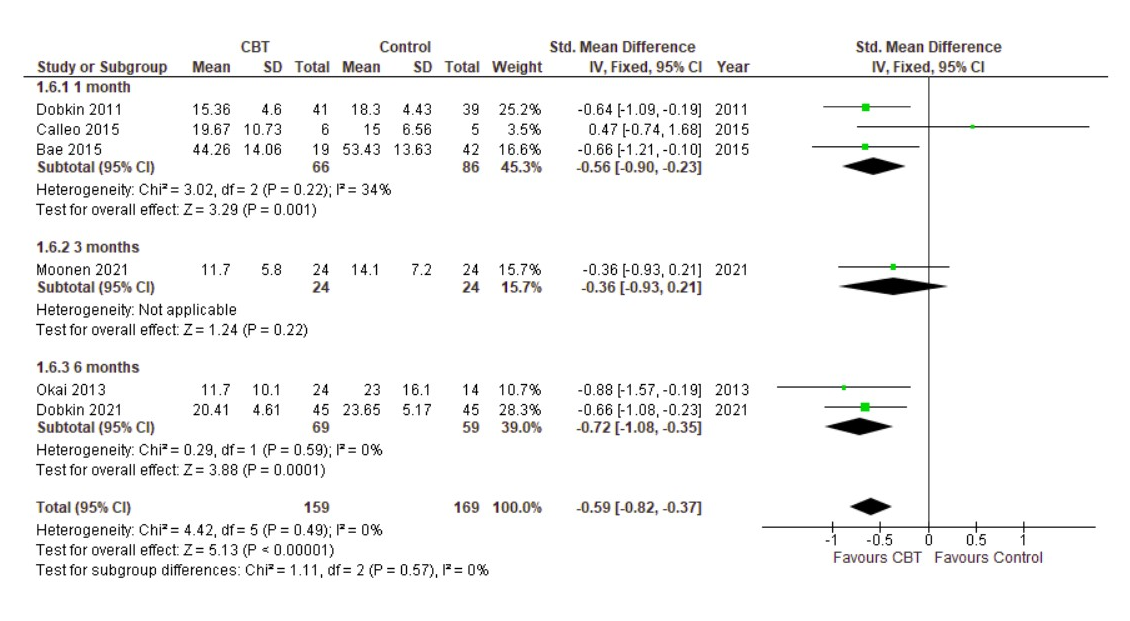
**supplementary material .5**: Forest plot of the long-term effects of CBT on Anxiety versus control in PD patients based on follow-up values of different time points.
